# Mixed Rigid and Flexible Component Design for High-Performance Polyimide Films

**DOI:** 10.3390/polym9090451

**Published:** 2017-09-15

**Authors:** Xiaohui Yu, Weihua Liang, Jianhua Cao, Dayong Wu

**Affiliations:** Technical Institute of Physics and Chemistry, Chinese Academy of Sciences, Beijing 100190, China; xhyu@mail.ipc.ac.cn (X.Y.); lwh@mail.ipc.ac.cn (W.L.); caojh@mail.ipc.ac.cn (J.C.)

**Keywords:** polyimide film, linear coefficient of thermal expansion (CTE), copper clad laminate, structure and properties

## Abstract

To develop the polyimide (PI) which is closely matched to the coefficient of the thermal expansion (CTE) of copper, a series of PIs are prepared from 5,4′-diamino-2-phenyl benzimidazole (DAPBI), 4,4′-diaminodiphenyl ether (ODA), and 3,3′,4,4′-benzophenonetetracarboxylic dianhydride (BTDA) using a sequential copolymerization, blade coating, and thermal imidization process. The physical properties of the PIs are effectively regulated and optimized by adjusting the ratio of the rigid DAPBI and flexible ODA components. By increasing the DAPBI content, thermal stability, dimensional stability, and mechanical properties, the resultant polymer is enhanced. PI-80 exhibits an excellent comprehensive performance, a glass transition temperature of 370 °C, and a tensile strength of 210 MPa. Furthermore, the CTE as calculated in the range 50–250 °C is ca. 19 ppm/K, which is equal to that of copper. A highly dimensionally stable, curl-free, and high T-style peel strength (6.4 N/cm) of copper/PI laminate was obtained by casting the polyamic acid onto copper foil (13 μm) and thermally curing at 360 °C, which indicates that it has the potential to be applied as an electronic film for flexible displays and flexible printed circuit boards. A structural rationalization for these remarkable properties is also presented.

## 1. Introduction

Aromatic polyimides (PIs) exhibit outstanding thermal stability, mechanical properties, solvent resistance, and electric insulation performance [1,2,3,4,5,6]. Aromatic PI films are widely used in the aerospace, automobile manufacture, and microelectronics fields owing to their facile fabrication by casting methods [7,8,9,10,11,12,13,14,15,16]. For example, PI films can be used as high-temperature-resistant insulating materials, flexible printed circuits, stress-relieving buffer layers, particle-blocking layers, dielectric and flexible connecting materials for multichip model systems, substrates for flexible solar cells or flexible displays, and lithium-ion battery separators [17,18,19,20,21].

Multichip modules require dielectric interlayers with low dielectric properties, excellent mechanical properties, and excellent adhesion. It is especially important that the coefficient of thermal expansion (CTE) of the interlayer should match that of the substrate (e.g., Si wafer, copper foil, aluminium foil) [22]. The use of PIs as dielectric layers can effectively enhance the safety and reduce the weight of electronic devices. However, typical commercial PI films can’t tolerate the high temperatures associated with welding fabrication. Furthermore, there is a significant difference in CTE between the PI films and common substrates. For example, the CTEs of copper, silicon wafer, and glass are 19, 6, and 15 ppm/K, respectively [23,24]. Additionally, the CTEs of the commercial PI films Kapton and Upilex without stretching are ca. 47 and 45 ppm/K, respectively [25]. 

The CTEs of polymers can be tuned by modulating their chemical structures and aggregation properties. There are three strategies commonly used to reduce the CTEs of PI films. The first approach is to subject the PAA or PI film to uniaxial or biaxial stretching at a high temperature (>150 °C) [26,27,28]. Upon stretching, the orientation of the molecular chains is improved along the stretching direction by promoting the aggregation of the PIs, and the regularity of the molecular chains is enhanced. However, the reduction of the CTE data by this method is limited owing to the small stretching ratios possible without causing permanent damage. Moreover, the PIs suitable for stretching treatment are typically thermoplastic and the glass transition temperature (*T*_g_) values of PIs are lower than 300 °C.

The second approach is to introduce inorganic nanoparticles that exhibit very low CTEs into the PI system in order to obtain the PI composite films with low CTEs [29,30,31,32]. The sol-gel method has been adopted, in which the precursors of inorganic particles are introduced into the polyamic acid (PAA) solution. However, macroscopic phase separation occurs in PI systems when the inorganic-particle content is higher than 5 wt %, and the properties of the resultant polymers are affected [33,34,35,36]. Geng et al. introduced reactive groups that can be linked with inorganic components into the PI backbone and adopted the end group (3-aminopropyl)triethoxysilane (APTES) to mitigate this macroscopic phase separation. However, this method had little impact on the CTEs of these inorganic-organic composite PI films. 

The third approach is to import the rigid rod-like, planar structures or short-chain diamines and dianhydride monomers into the PI unit structure to enhance the proportion of rigid benzene rings and amide rings. This method can reduce the CTEs of PI films because it promotes the ordered arrangement and degree of molecular orientation in the polymer chains. Previous researches have suggested that designing polymers with rigid and ordered molecular chains is the best way to improve their thermal and dimensional stability [37,38,39,40].

Significant research efforts have been devoted to developing high-performance PIs comprising imidazole and oxazole rings owing to the outstanding heat resistance, high strength, and high moduli of polybenzimidazoles and polybenzoxazoles [41,42,43]. However, the flexibility of these films is poor owing to the high rigidity of the rod-like chains. Furthermore, the CTEs of these films are often negative, which mismatches those of common substrate materials [37,43].

To obtain a PI film with an equal CTE to that of copper over a wide temperature range, a series of different structural PIs were synthesized and optimized. The introduction of both rigid and flexible molecular units into polymer chains is an effective way to control and optimize their properties [37]. Consequently, in this study, PI films were prepared using the structurally rigid diamine 5,4′-diamino-2-phenyl benzimidazole (DAPBI), the structurally flexible diamine 4,4′-diaminodiphenyl ether (ODA), and 3,3′,4,4′-benzophenonetetracarboxylic dianhydride (BTDA). PIs containing different percentages of ODA–BTDA and DAPBI–BTDA chain segments were synthesized by copolymerizing the diamines ODA and DAPBI with BTDA. ODA and BTDA, which exhibit flexible structures, were chosen to provide flexibility to the polymers. The rigid structural diamine DAPBI provides heat resistance and excellent mechanical properties to the polymers. By optimizing the ratio of flexible ODA–BTDA segments and rigid DAPBI–BTDA segments, the flexibility, CTE, mechanical properties, and thermal properties of PIs were tuned. When the ratio of ODA-BTDA and DAPBI-BTDA is 8:2, the optimized PI has a CTE value close to that of copper, and it also exhibits outstanding heat resistance and mechanical properties. 

## 2. Materials and Methods 

### 2.1. Materials

*N*,*N*-Dimethylacetamide (DMAc) was obtained from Tianjin Chemical Agent Factory (Tianjin, China) and was vacuum-distilled after drying over calcium hydride. BTDA and ODA were purchased from Forsman Scientific Company (Beijing, China). DAPBI was purchased from Nanjing Chemlin Chemical Industry Company (Nanjing, China). The dianhydride was dried in a vacuum oven at 150 °C for 12 h and the diamines were dried at 80 °C for 12 h. To improve the relative molecular weight, all the reaction vessels, solvents, and reactants must be anhydrous.

### 2.2. Methods

#### 2.2.1. Infrared Spectroscopic Analysis

The inherent viscosities (η) of the PAAs were measured to evaluate the polymerization degree of the PIs. η was obtained by dissolving 0.5 g of PAA in 100 mL DMAc at 25 °C, and the flow of the PAA solution was measured using an Ubbelohde viscometer (Zhongwang Technology, Hangzhou, China). The η value was calculated using the following equation:

(1) $$\eta =\frac{\ln\left(t/t_{o}\right)}{0.5\;g/\mathrm{dL}}$$

where *t* and *t*_0_ are the determination times for the PAA solution and a blank DMAc solution, respectively. In order to evaluate the imidization degree of the PI films, their Fourier transform infrared (FTIR) spectra were obtained on a Excalibur 3100 spectrometer (Varian, Palo Alto, CA, USA) from 600 to 4000 cm^−1^.

The equilibrium water absorbability was determined by the weight difference of vacuum dried film specimens before and after immersion in deionized water at 25 °C for 24 h. Dry films which had been weighed (*W*_1_) were immersed in water at room temperature for 24 h. The surfaces of the films were wiped to remove the water and weighed again (*W*_2_). Water absorption was calculated by the equation: (*W*_2_ − *W*_1_)/*W*_1_ × 100%.

#### 2.2.2. Morphology and Structural Analysis

The microstructures of the polymers were characterized on Rigaku wide-angle and small-angle X-ray diffractometers (Rigaku Corporation, Tokyo, Japan). The 2θ scan data were collected at 0.02° intervals over ranges of 5°–60° and 0.6°–5°. The scan speed was 0.1°/min. The average distance (d) between the molecular chains can be obtained from Bragg’s equation:

d = λ/2sin θ (2)

where 2θ is the X-ray diffraction angle and λ is the wavelength of the X-rays. The cross-sectional micromorphologies of the PI films were sputtered with gold and then investigated using scanning electron microscopy (SEM) (Hitachi Limited, Hitachi, Japan) with HITACHI S-4300-4300 apparatus at an acceleration voltage of 10 kV. Moreover, the surface morphology and the element mapping of the PI layer and copper substrate after the T-peel test were measured with SEM and Energy Dispersive X-ray Spectrometric microanalysis (EDX) (Hitachi Limited, Hitachi, Japan) with HITACHI S-4300 apparatus.

#### 2.2.3. Thermal Analysis

In order to investigate the thermal properties, the glass transition temperature (*T*_g_), thermal decomposition temperature (*T*_d_), and the CTEs of the films were characterized. The *T*_g_ values of the PI films were determined on a Mettler Toledo DSC 1 thermal analyzer (METTLER TOLEDO, Zurich, Switzerland). DSC measurements were carried out on 5–10 mg film samples heated under nitrogen atmosphere at a heating rate of 10 °C/min from 50 to 400 °C. The samples were first heated from 50 to 400 °C, then cooled at the same heating rate, and then heated again at the same conditions. The *T*_g_ of these PIs were determined in the second heating scan by the midpoint ASTM D3417-83 with the Mettler software. Dynamic mechanical analysis (DMA) was performed on thin film specimens (ca. 3 × 0.65 × X cm^3^, the X represent the thickness of the film, which is about 3 × 10^−3^ cm) on a TA instrument DMA Q800 (TA Instruments, New Castle, PA, USA) at a heating rate of 5 °C/min and at a load frequency of 1 Hz under nitrogen atmosphere. The peak on the tan δ vs. temperature curve was regarded as the *T*_g_ of the film. *T*_d_ values for the PI films were obtained on a TA Q50 thermogravimetric analyzer (TA Instruments, New Castle, PA, USA). Measurements were carried out on 3–5 mg film samples heated at 5 °C/min in nitrogen atmosphere. Decomposition temperatures of 5% weight loss were recorded (*T*_d5%_) to evaluate the thermal stability of the films. The CTEs of the films were measured using a Mettler Toledo TMA/SDTA841^e^ (METTLER TOLEDO, Zurich, Switzerland). A constant force of 0.01 N was applied to film samples (ca. 1.5 × 0.4 × X cm^3^). The samples were heated to 400 °C at a heating rate of 5 °C/min. The data were collected from the second heating run after the first run, which was performed up to the annealing temperature to eliminate the internal stresses in the films. Owing to the variation of the CTE with temperature, it is common practice to report the average CTEs determined over a specified temperature range. The average CTE is defined by:

(3) $${\bar{a}}_{L}=\frac{1}{L_{0}}\left(\frac{\Delta L}{\Delta T}\right)$$

where *L*_0_ is the initial length of the sample between the grips at ambient temperature; Δ*T* = *T*_2_ − *T*_1_, where *T*_1_ and *T*_2_ are the temperature limits; and Δ*L* = *L*_2_ − *L*_1_, where *L*_1_ and *L*_2_ are the sample lengths at temperatures *T*_1_ and *T*_2_, respectively. All of the CTEs in this study were measured between 50 and 250 °C in the film plane direction and were derived using Equation (3).

#### 2.2.4. Mechanical Analysis

The tensile properties of the films were examined with an Instron R5966 (INSTRON corproration, Canton, OH, USA). Tensile samples were machined to dimensions of ca. 4 × 0.5 × X cm^3^ after imidization and tested with a crossed-head speed of 8 mm/min and a load capacity of 500 N. Each test was conducted on five samples, and the average tensile strength, modulus, and elongation at the break are reported.

#### 2.2.5. Adhesion Properties 

The adhesion strength between the PI layer and copper substrate was studied by the T-peel strength test method with an Instron R5966 (INSTRON corproration, Canton, OH, USA). The laminate samples were cut into 5 cm × 0.5 cm strips. The peeling speed was kept constant at 10 mm/min. The peel strength was expressed in N/cm and was determined from the peel load divided by the width of the PI/Cu laminate.

#### 2.2.6. Synthesis of PAA Precursors

PAA precursors were prepared by copolymerization of the diamines ODA and DAPBI and the dianhydride BTDA at a 1:1 molar ratio of diamines and dianhydride. Six different PAAs were obtained by employing diamine mixtures with DAPBI molar contents of 0%, 20%, 40%, 60%, 80%, and 100%. The corresponding PI films were fabricated by coating, solvent drying, and thermal imidization, and denoted as PI-0, PI-20, PI-40, PI-60, PI-80, and PI-100. 

The polymerization procedure for synthesizing the PAA for the PI-60 precursor is presented as an example: The molar ratio of ODA to DAPBI was 4:6, and the solid content was controlled at 15%. First, the ODA (1.602 g, 8 mmol) was dissolved in 60 mL DMAc at room temperature under N_2_ atmosphere. After the ODA was dissolved, DAPBI (2.691 g, 12 mmol) was dispersed uniformly in the ODA solution, and a beige brown suspension was obtained. Then, a stoichiometric amount of BTDA (6.445 g, 20 mmol) was added gradually to the suspension, and the mixture was stirred continually for 6 h to form a viscous PAA precursor solution. The PAA was sealed and stored at 0 °C. 

#### 2.2.7. Thermal Imidization of PAA Films

PI films were obtained by casting the PAA solution onto clean, dry glass plates with a 300 μm depth blade. The solvent was then removed by drying the PAA films in an oven with a programmed procedure (50 °C for 1 h, 80 °C for 1 h, 100 °C for 1 h, 120 °C for 0.5 h, 150 °C for 0.5 h, and 180 °C for 0.5 h) at a heating rate of 10 °C/min. The PAA films were then cured in a vacuum oven at a heating rate of 5 °C/min to afford PI films. The PI-0 film was obtained with a curing program of 200 °C for 1 h, 250 °C for 1 h, 300 °C for 0.5 h, and 360 °C for 0.5 h. All the cured films were cooled to room temperature at a cooling rate of 3 °C/min. The PI films with thicknesses of 30 μm were easily peeled from the glass plate.

## 3. Results

### 3.1. Synthesis of PAAs and Fabrication of PI Films

**Scheme 1:**
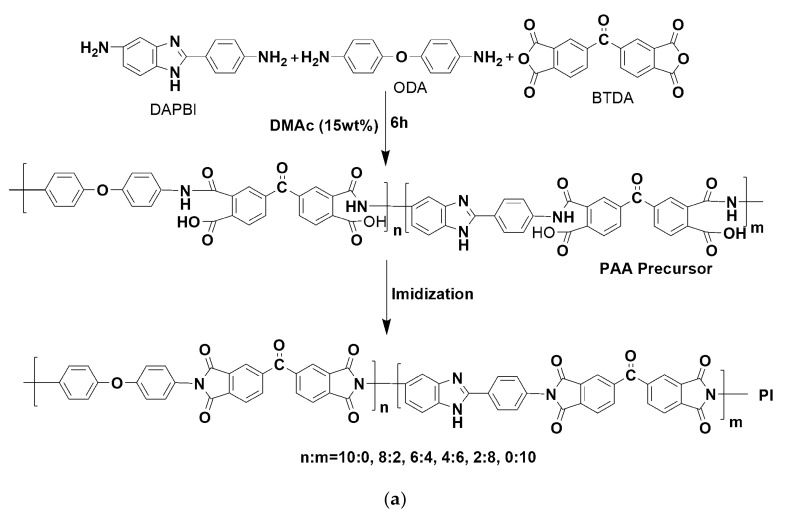

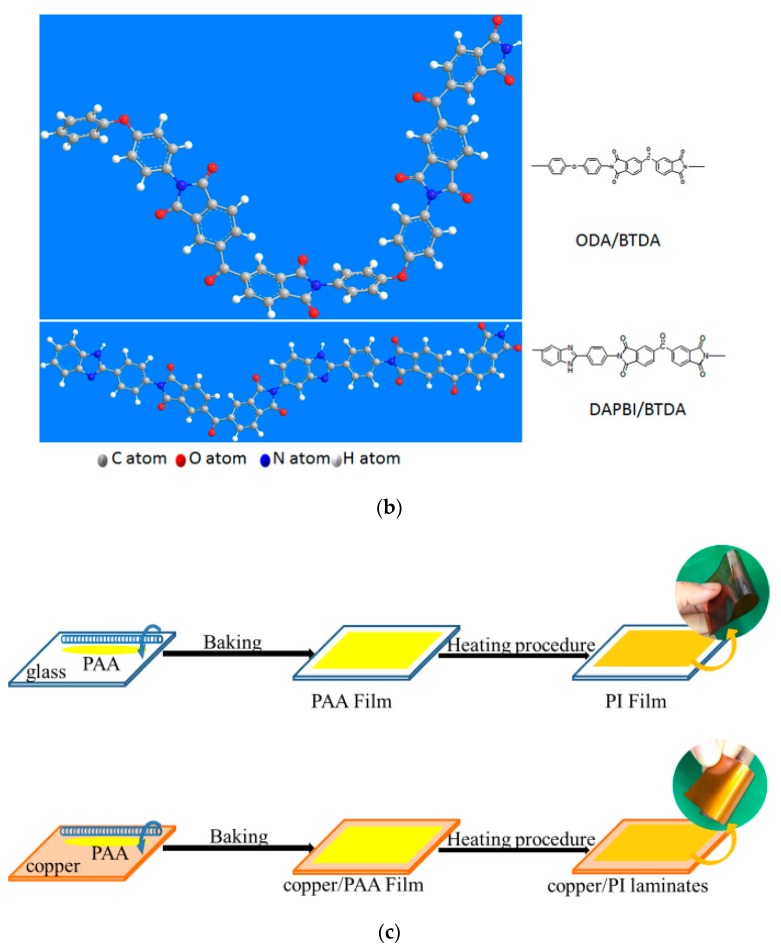
(**a**) Synthesis of PAAs and thermal imidization of PIs. (**b**) Chemical structure of the homo-PI dimers ODA/BTDA and DAPBI/BTDA. (**c**) Fabrication of PI films and PI/copper laminates.

The PI films containing benzimidazole moieties were obtained by the polymerization of the PAA precursor, blade coating, baking, and thermal imidization processes. The synthesis of the PIs, the molecular modeling of a homo-PI dimer simulated by ChemDraw (Cambridge Soft, Waltham, MA, USA), and a schematic diagram of the procedure for preparing the PI films and PI/copper laminates are shown in Scheme 1. PIs containing different ratios of the flexible ODA–BTDA segment and the rigid DAPBI–BTDA segment were prepared by adjusting the ratio of two diamines employed in the synthesis. Molecular models of the homo-PI ODA–BTDA and DAPBI–BTDA dimer structures were generated using ChemDraw software, and those with the lowest energy conformation are shown in Scheme 1b. The simulated results show that the ODA–BTDA dimer adopts a bent conformation and the DAPBI–BTDA dimer exhibits a planar construction. Hence the flexible ODA–BTDA segment governs polymer flexibility, and the DAPBI–BTDA segment endows the polymer chains with rigidity and regularity. Furthermore, the intermolecular hydrogen bonding formed between the N–H groups in DAPBI and the carbonyl groups of the imide ring can increase the intermolecular interaction strength. Thus, the DAPBI–BTDA segment was selected to improve the mechanical properties, dimensional stability, and thermal stability of the PIs [22]. According to the literature, benzimidazole moieties can react with copper oxide to form copper imidazolate inner complexes that provide active sites for coordinating interfacial bonding. Furthermore, the PIs containing the ODA diamine can exhibit improved copper adhesion owing to their flexible structures [44,45].

### 3.2. Viscosities of PAAs and FTIR Analysis of the PI Films

**Figure 1:**
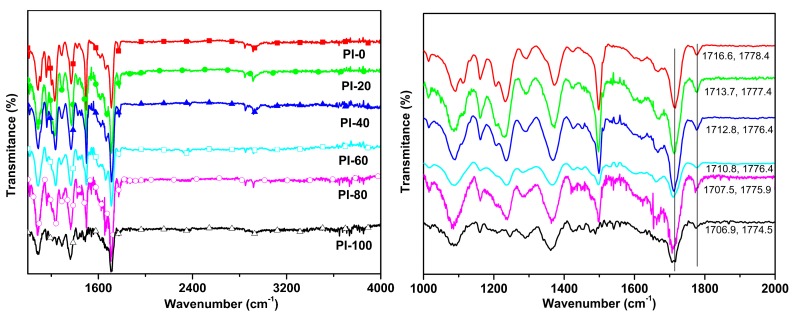
FTIR spectra of PI films.

**Figure 2:**
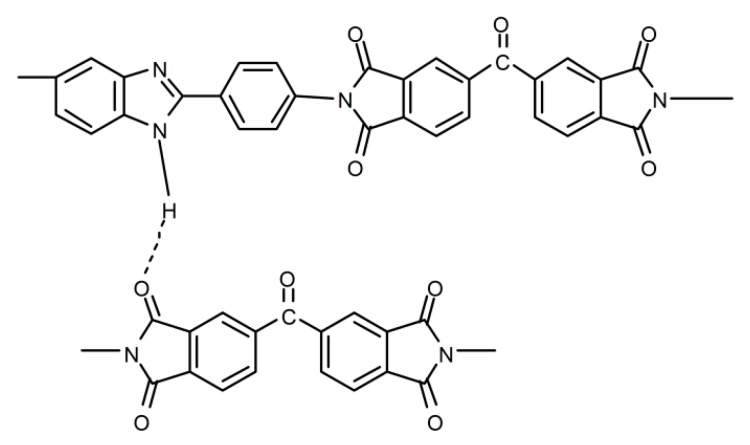
Interchain hydrogen bonding.

As the polymerization degree of the PAAs and imidization degree of the PIs affect the thermal properties and mechanical properties of the PI films, both polymerization and imidization were evaluated. First, the inherent viscosities of the PAAs were measured at 25 °C to assess their degree of polymerization, and the results are listed in Table 1. The high viscosities (1.8–2.3 dL/g) indicate that the PAAs obtained have moderately high molecular weights. The FTIR spectra of the PI films were recorded to assess the degree of imidization and they are shown in Figure 1. The characteristic absorption peaks of the symmetrical contraction vibration and the antisymmetric stretching vibration of the carbonyl group in the imide ring are observed at 1778 and 1716 cm^−1^, respectively. Furthermore, the PI films present no absorption peaks characteristic of carboxyl and amide linkages, i.e., at 3500 cm^−1^ (COOH and NH), 1660 cm^−1^ (CONH), and 1550 cm^−1^ (C–NH), indicating that the PI films have undergone complete imidization. Upon increasing the molar percentage of benzimidazole stepwise from 0% to 100%, the carbonyl absorption peaks gradually shift from 1778.4 and 1716.6 cm^−1^ to 1774.5 and 1706.9 cm^−1^, respectively. This indicated that hydrogen bonds form between the carbonyl group of the imide ring and the NH group of DAPBI. Thus, more hydrogen bonds may be formed with an increased imidazole ring content, and these intermolecular forces cause the carbonyl absorption peaks to move to a lower wavenumber [46,47]. A diagram showing this intermolecular hydrogen bonding is shown in Figure 2. All obtained films exhibited excellent chemical resistance and were insoluble in organic solvents, for example, NMP (*N*-Methyl-2-Pyrrolidone), m-cresol, DMAc, chloroform, etc. The water absorption results of these films are listed in Table 1. Owing to the existent intermolecular hydrogen bonding, the water absorption is a little higher than common polyimides.

### 3.3. Morphology and Structures

**Figure 3:**
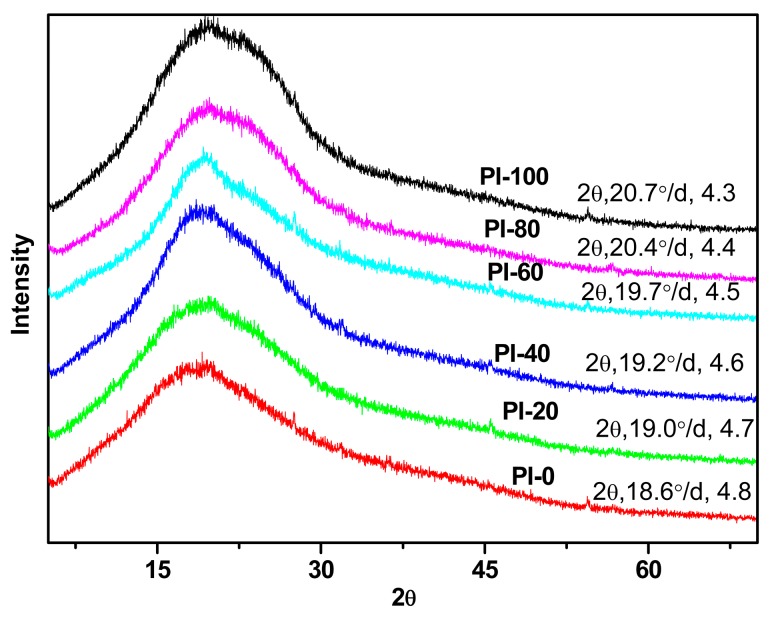
WAXD patterns of the PI films.

The wide-angle X-ray diffraction (WAXD) patterns of the PI films are shown in Figure 3. Each presents a broad diffraction peak without obvious splitting peaks, indicating that the PIs are essentially amorphous. With an increase in the DAPBI–BTDA content, narrower and stronger intensity diffraction peaks with slight splitting are observed, indicating that the regularity of the molecular chains and the order degree of molecular chains are improved. According to Bragg’s law (see Equation (2)), the distance between the molecular chains decreased from 4.8 to 4.3 Å. This demonstrates that the intermolecular interactions increase when the benzimidaole content increases.

**Figure 4:**
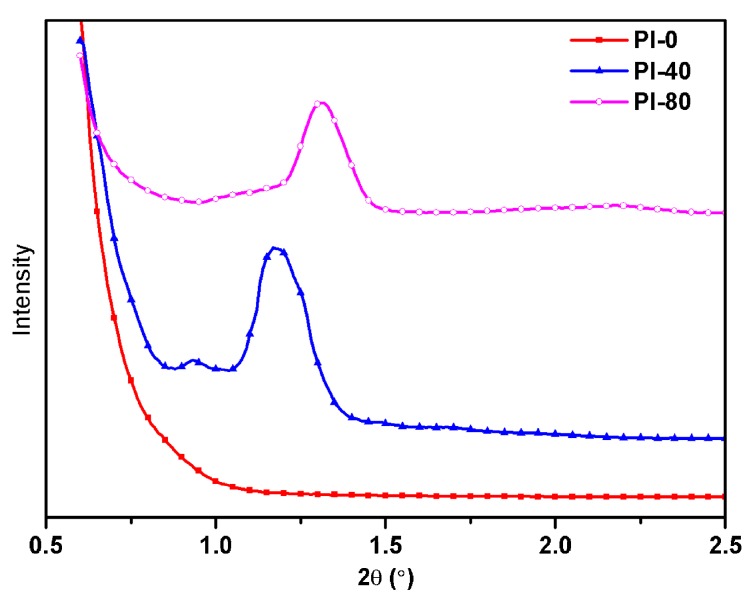
SAXD patterns of the PI films.

Figure 4 shows representative small-angle X-ray diffraction (SAXD) patterns for the PI-0, PI-40, and PI-80 films. The PI-0 sample presents no SAXD peaks because the polymer comprises a completely flexible and disordered molecular chain. Upon the introduction of DAPBI into the molecular chains, an SAXD diffraction peak appears at close to 1.2°, and the angle increases with increasing DAPBI content. According to Bragg’s law (see Equation (2)), the long period of PI-40 and PI-80 is 75.4 and 65.4 Å, respectively. The SAXD results indicate that local structure ordering occurs in the PIs containing benzimidazole moieties, and the period diminishes with increasing benzimidazole content. This shows that the molecular chains containing benzimidazole rings possess a certain degree of planarity and rigidity, and thus, local structural ordering appears and the regularity of the molecular chain is improved as the DAPBI molar ratio increases.

**Figure 5:**
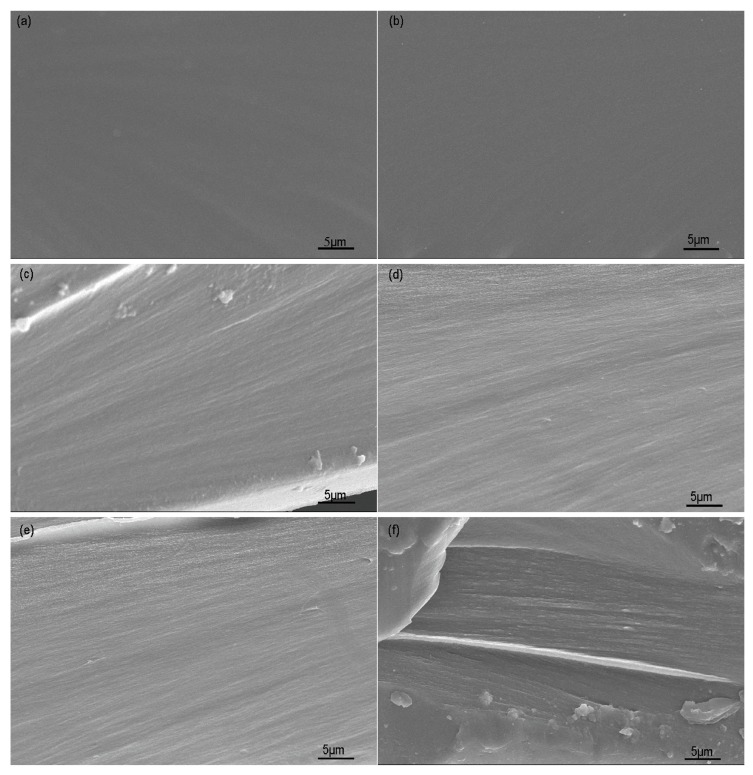
Cross-sectional SEM images of the PI films. (**a**) PI-0; (**b**) PI-20; (**c**) PI-40; (**d**) PI-60; (**e**) PI-80; (**f**) PI-100.

The cross-sectional morphologies of the PI films were observed by SEM, as shown in Figure 5. The morphologies of films PI-0 and PI-20 are smooth. However, when the DAPBI molar percentage is higher than 40%, densely packed layered ribbon-like morphological features appear. This indicates that the molecular chains of the polymers have poor regularity when the benzimidazole content is low. Conversely, the molecular chains of the polymer are arrayed more regularly, and the degree of orientation and local ordering improves with the influence of the benzimidazole component [41].

### 3.4. Thermal Properties

**Figure 6:**
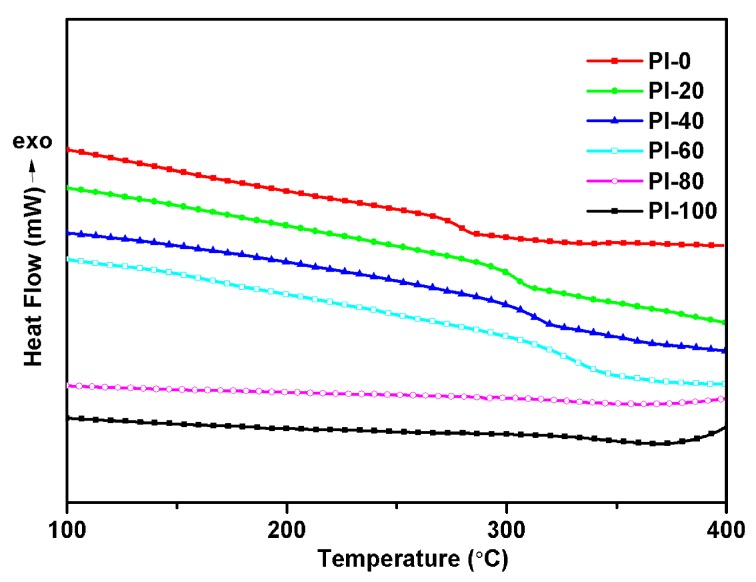
DSC curves of PI films.

**Figure 7:**
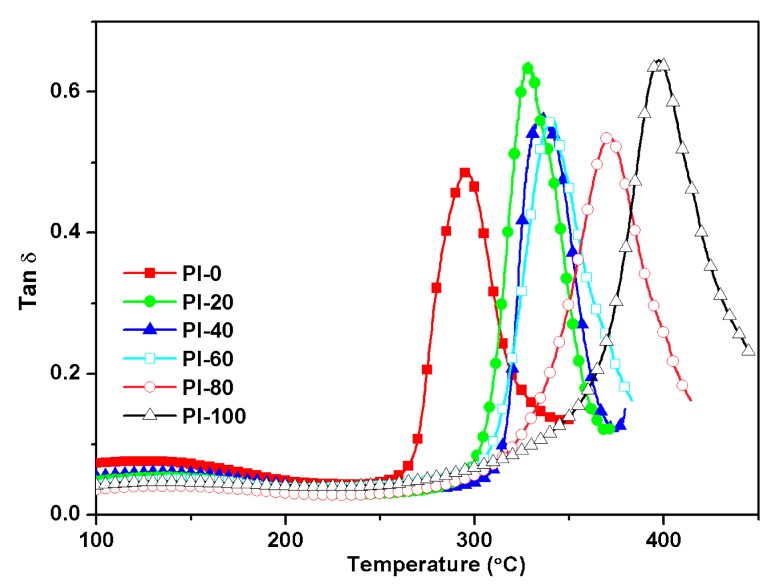
Tan δ vs. temperature for the PI films.

**Figure 8:**
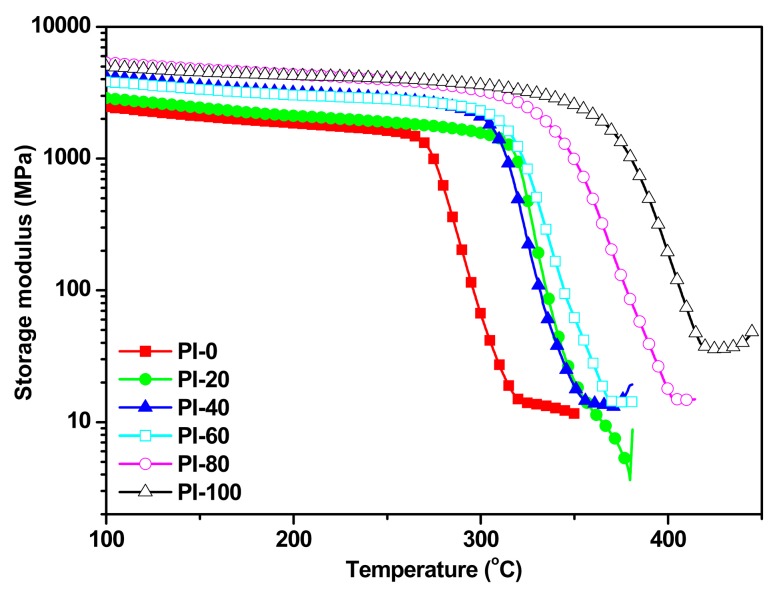
Storage modulus as a function of temperature for PI films.

The *T*_g_, *T*_d_, and CTE data are given in Table 1. The DSC curves of films are shown in Figure 6. No endothermic peaks assigned to the melting peak are observed in the DSC curves, indicating that all the PIs are amorphous polymers. Owing to the poor molecular mobility introduced by the rigid structures, no well-defined *T*_g_ is detected for PI-80 and PI-100. The tan δ curves and storage modulus curves of films as obtained by DMA are shown in Figure 7 and Figure 8. The *T*_g_ data (Table 1) obtained from DSC and DMA are in the range of 278–400 °C, indicating that these PIs exhibit high heat resistance. With increasing DAPBI content, the rigidity of the polymer chains is enhanced, so the *T*_g_ of the PIs increases. The storage modulus curves show that all the films have large storage moduli (3–6 GPa) at 100 °C and still retain moduli of 10–100 MPa at temperatures above 300 °C. This demonstrates that the PIs with a higher content of DAPBI are very hard materials.

**Figure 9:**
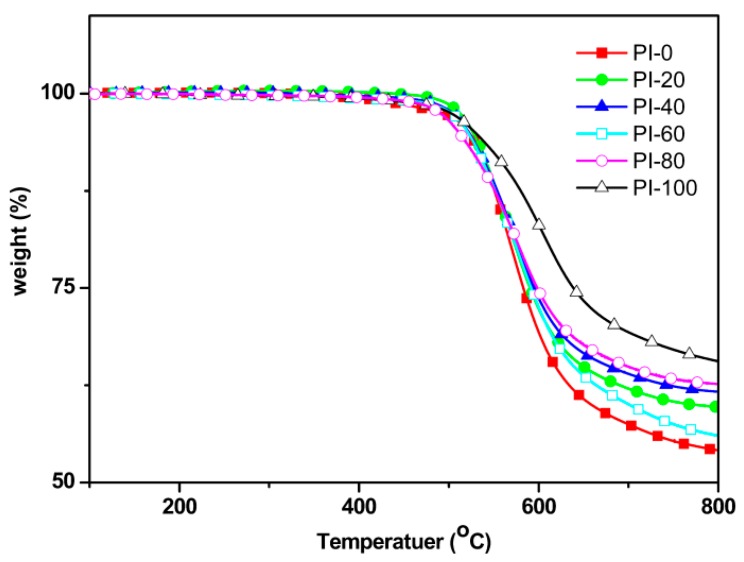
TGA curves of the PI films.

The thermogravimetric analyses (TGA) of the PI films are shown in Figure 9 and show that the films present no weight loss below 480 °C. The excellent thermal stability indicates that these PI films are completely imidized. The films exhibit 5% weight loss in the temperature range of 521–529 °C in N_2_, and the char yield at 800 °C reaches 65%. 

**Figure 10:**
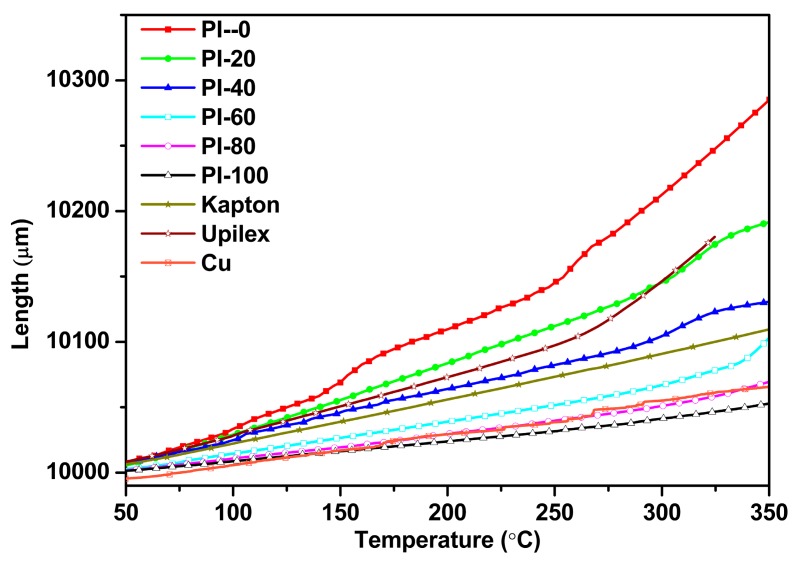
CTE curves of the PI films.

The temperature-strain curves of the films, commercial polyimides, and pure copper are shown in Figure 10. The average CTEs as calculated in the ranges 50–250, 50–300, and 100–200 °C are listed in Table 2. The curves in Figure 10 show that the strain of the films increases with an increasing temperature and the PI-0 and PI-20 increase more rapidly. This is mainly because the molecular chains of these two polymers are flexible, and the films deform far more rapidly at a high temperature. Films with a benzimidazole content over 40% exhibit increasingly excellent thermal dimensional stabilities than commercial polyimides, and the CTEs are less than 40 ppm/K. The temperature-strain curve of PI-80 is identical to the pure copper’s temperature-strain curve. With the rigid structural DAPBI content increased, the regularity of molecular chains is enhanced. Thus, the CTE data of films decrease, namely, the dimensional stability is improved. 

### 3.5. Adhesion Properties of PI-80/Copper Laminate

**Figure 11:**
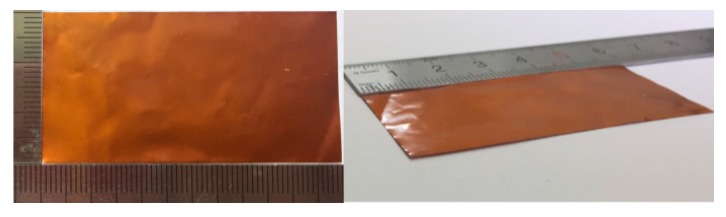
PI/copper laminates with PI layer thicknesses of 20 μm.

**Figure 12:**
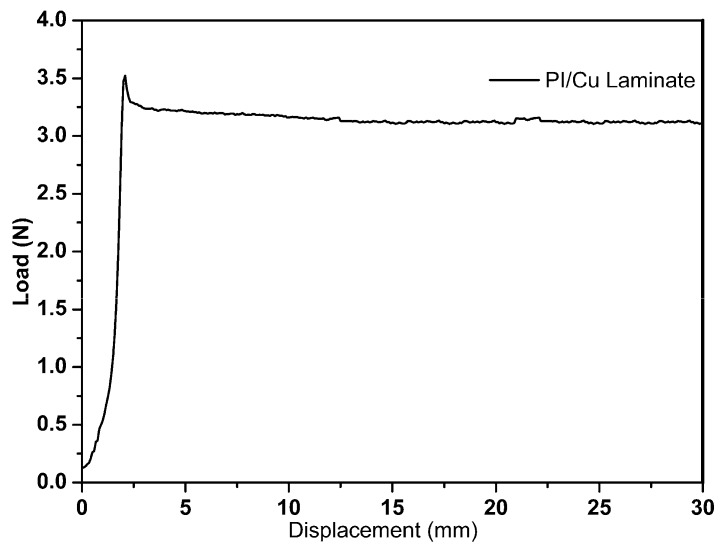
The load-displacement curve of the PI/Cu laminate.

**Figure 13:**
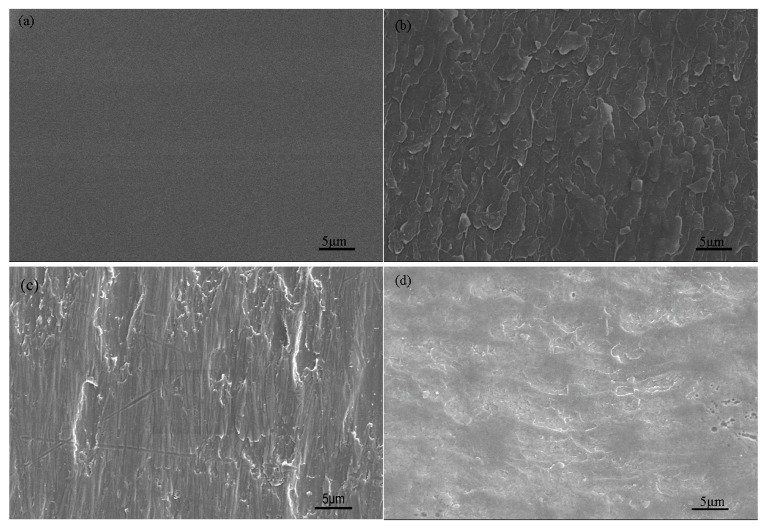
Surface SEM images of the PI-80 and copper substrate. (**a**) PI-80 film without any treatment; (**b**) PI film of PI-80/Cu laminate after peeling; (**c**) copper without any treatment; (**d**) copper substrate of the PI-80/Cu laminate after peeling.

**Figure 14:**
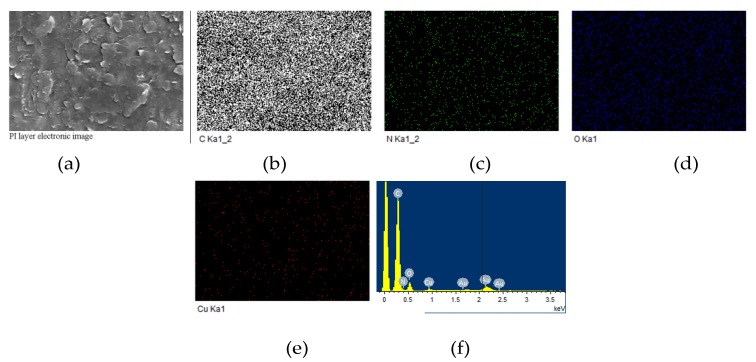
The element mappings of the peeled surface of PI-80 layer. (**a**) electronic image of PI layer; (**b**) C element in PI layer; (**c**) N element in PI layer; (**d**) O element in PI layer; (**e**) Cu element in PI layer; (**f**) EDX spectrum of peeled PI layer.

**Figure 15:**
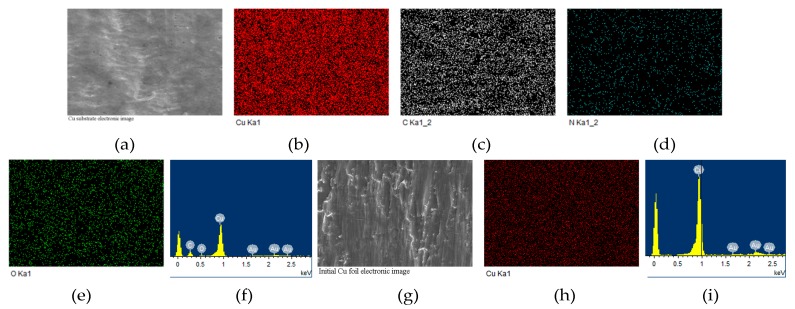
The element mappings of the peeled surface of copper substrate and initial Cu foil. (**a**) electronic image of Cu substrate; (**b**) Cu element in Cu substrate; (**c**) C element in Cu substrate; (**d**) N element in Cu substrate; (**e**) O element in Cu substrate; (**f**) EDX spectrum of peeled copper substrate; (**g**) electronic image of initial Cu foil; (**h**) Cu element on initial Cu foil; (**i**) EDX spectrum of pure Cu foil.

**Figure 16:**
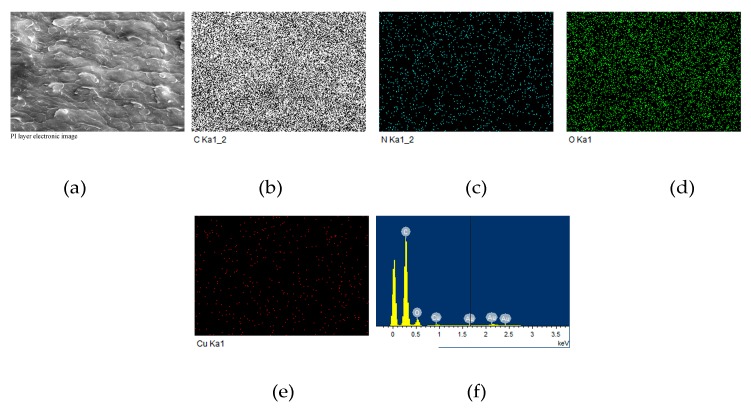

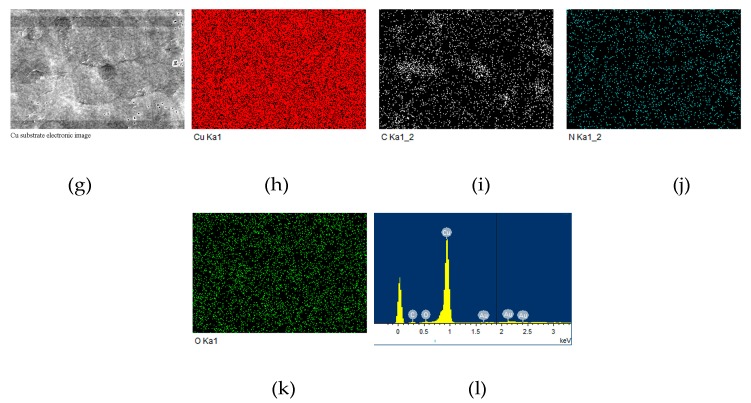
The element mappings of the peeled surface of PI-60/copper laminate. (**a**) electronic image of PI-60 layer; (**b**) C element in PI layer; (**c**) N element in PI layer; (**d**) O element in PI layer; (**e**) Cu element in PI layer; (**f**) EDX spectrum of peeled PI layer; (**g**) electronic image of Cu substrate; (**h**) Cu element in Cu substrate; (**i**) C element in Cu substrate; (**j**) N element in Cu substrate; (**k**) O element in Cu substrate; (**l**) EDX spectrum of Cu substrate.

As shown in Figure 10 and Table 2, the PI-80 film has a comparable CTE to that of pure copper for a wide temperature range. The equivalent CTE of copper and the PI film can avoid the internal stresses of the aggregation generated during the processing. Consequently, the PAA precursor of PI-80 was coated onto 13-μm-thick pure copper (purchased from LingBao JinYuan ZhaoHui Copper Co., Ltd, Lingbao, China, and without any pretreatment) and then thermally imidized under N_2_ atmosphere. The top-view and side-view photos of PI-80/copper laminates with PI layer thicknesses of 20 μm are shown in Figure 11. After being cured at 360 °C, the PI-80/copper laminates are flat and not warped. The adhesion strength between the PI layer and the copper substrate was tested by the T-peel strength test method with the PI-80/copper laminates. The load-displacement curve is shown in Figure 12. The peel strength is stabilization and the data is about 6.4 N/cm (3.2 N/0.5 cm), which indicated that a high enough adhesion laminate was obtained. The surface morphologies of the PI layer and copper substrate after peeling are shown in Figure 13. The surface of the pure PI-80 film without any treatment is smooth. The surface morphology of the PI-80 layer (b) after peeling becomes rough and displays a sheet structure. Also, the surface morphology of the copper substrate is different from the pure copper foil. It seems that there is one layer of polymers formed on the copper substrate. Further analysis of the interfacial interaction between the PI and copper substrate was conducted by Energy Dispersive X-ray Spectrometric microanalysis (EDX) with SEM. The element mapping and EDX spectrums of the peeled surface of PI-80 layer, copper substrate, and initial Cu foil are shown in Figure 14 and Figure 15. As shown in Figure 14, there is a Cu element on the PI layer beyond the C, N, and O elements. Additionally, there are C, N, and O elements on the copper substrate except for the Cu element (Figure 15a–f). But there are no C, N, or other elements on the initial Cu foil (Figure 15g–i). All the SEM and EDX results declare that there is a strong interfacial interaction between the PI and copper. The N-H in the imidazole group of DAPBI can react with copper oxide at the interface and also the flexible structural ODA improves the adhesion force. Therefore, the PI-80/copper laminates show excellent peel strength. The element mappings and EDX spectrums of the peeled surface of the PI-60/copper laminate scanned for an approximately equal time with PI-80/copper are shown in Figure 16. The results show that there is an interfacial interaction between the PI-60 and Cu foil. However, the smaller peeled sheets of the PI-60 layer and lower C element on the copper substrate indicate that the interfacial interaction between the PI-60 and copper is smaller. 

### 3.6. Mechanical Properties

**Figure 17:**
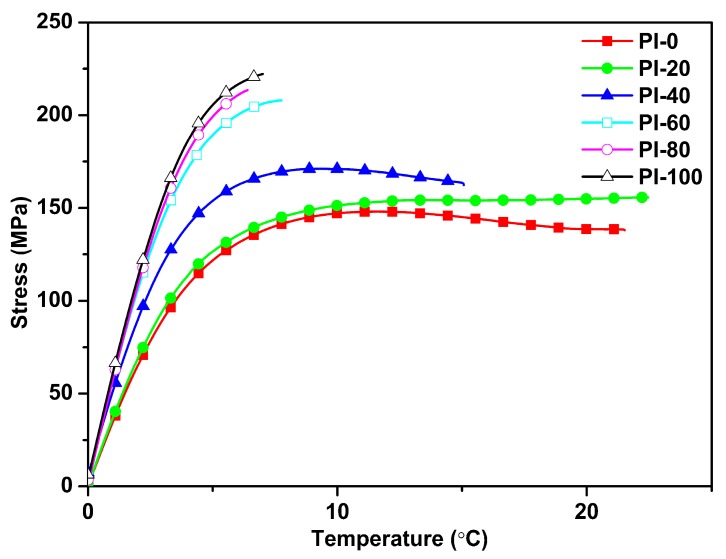
The stress-strain curves of PI films.

The mechanical properties of the PI films are listed in Table 3. All the films exhibit remarkable tensile strengths and moduli, which are in the ranges 146.0–220.8 MPa and 3.4–6.2 GPa, respectively. With increasing DAPBI content, the tensile strengths and moduli of the films increase, and their elongations decrease. The tensile strength of the PI-100 film is 1.5 times that of PI-0. The stress-strain curves of PI films are shown in Figure 17, showing that these PIs are amorphous polymers. The stress-strain curves of PI-0, PI-20, and PI-40 indicated these three polymers are hard and flexible materials. The stress-strain curves of PI-60, PI-80, and PI-100 declare that these three polymers are hard and tough materials. The results of tensile curves agree well with the DMA properties. The tensile results show that the rigid structural DAPBI could improve the rigidity and regularity of the polymer chains. According to the literature, the tensile strengths of the commercial films Kapton (PMDA-ODA) and Upilex-s (BPDA-ODA) without stretching are 130 and 140 MPa, respectively [25]. These PI films exhibit more outstanding mechanical properties, because of the rigid structure and stronger molecular interchain interaction.

## 4. Conclusions

To obtain a PI film with an equal CTE to that of copper, PIs containing flexible ODA–BTDA segments and rigid DAPBI–BTDA segments were prepared from ODA, DAPBI, and BTDA. Six different PI films with different molecular compositions were obtained by adjusting the molar ratio of the two diamines. Owing to the inclusion of rigid structural benzimidazole moieties and hydrogen bonds forming between the carbonyl group of the imide ring and the NH group of DAPBI, the regularity of the molecular chain improves as the DAPBI molar ratio increases. Hence, the PIs containing benzimidazole moieties exhibit excellent thermal stability, mechanical properties and dimensional stabilities. The *T*_g_ of the PI was enhanced to 400 from 280 °C, the CTE (50–250 °C) was reduced to 15 from 67 ppm/K, and the tensile strength was increased to 220 from 146 MPa. Furthermore, the CTE of the PI-80 film was found to be equal to that of copper and was therefore used to prepare a PI/copper laminate that exhibited no distortion or delamination when heated. The SEM and EDX results of the peeled PI/copper laminate interface reveal that a strong interfacial interaction formed between the PI and copper owing to the chemical nature of DAPBI and flexible structural ODA. The excellent flatness, folding resistance, and high peel strength of this PI/copper laminate indicate that this PI film has potential for use in flexible printed circuit boards, flexible display substrates, and other next-generation electronic devices. 

## Figures and Tables

**Table 1:** Inherent viscosities and thermal properties of the PI films.

Samples	η_inh_^1^ (dL/g)	*T*_g_^2^ (°C)		*T*_d5%_^3^ (°C)	R_800_^4^ (%)	Water absorption (%)
		DSC	DMA			
PI-0	1.85	278	296	523	54.2	2.0
PI-20	2.06	307	328	527	59.7	2.3
PI-40	2.20	314	334	522	61.7	2.5
PI-60	2.25	343	340	521	56.1	2.9
PI-80	2.30	NF ^5^	371	523	62.7	2.5
PI-100	2.15	NF	397	529	65.6	3.0

^1^ Inherent viscosity determined with a concentration of 0.5 g/dL in DMAc at 25 ± 0.1 °C. ^2^ Glass transition temperature (*T*_g_) measured by DSC and DMA at a heating rate of 10 and 5 °C/min, respectively. ^3^ Temperatures at 5% weight loss were recorded by TGA at a heating rate of 5 °C/min under nitrogen atmosphere. ^4^ Residual weight retention at 800 °C under nitrogen. ^5^ Not found.

**Table 2:** CTE data for PI films in different temperature ranges.

Samples	CTE (50–250 °C) ppm/K	CTE (50–300 °C) ppm/K	CTE (100–200 °C) ppm/K
PI-0	67.9	82.0	76.2
PI-20	53.3	55.8	54.5
PI-40	37.8	38.6	38.9
PI-60	24.4	25.6	24.4
PI-80	19.0	19.6	18.8
PI-100	15.2	15.9	15.4
Cu	19.0	19.4	18.6
Kapton	33.5	34.2	33.0
Upilex	44.9	55.3	43.4

**Table 3:** Mechanical properties of PI films.

Samples	Elastic (GPa)	Max stress (MPa)	Break strain (%)
PI-0	3.4 ± 0.05	146.0 ± 1.9	16.6 ± 4.9
PI-20	3.8 ± 0.14	157.8 ± 3.4	21.5 ± 8.7
PI-40	4.9 ± 0.16	173.3 ± 4.8	9.4 ± 3.8
PI-60	5.8 ± 0.07	205.6 ± 8.8	7.3 ± 1.0
PI-80	5.7 ± 0.48	209.0 ± 5.9	6.2 ± 0.4
PI-100	6.2 ± 0.37	220.8 ± 10.0	6.8 ± 1.2
